# Identifying Knowledge Gaps in Individuals With Primary Adrenal Insufficiency: A Critical Step in Preventing Adrenal Crisis

**DOI:** 10.1111/cen.70006

**Published:** 2025-07-22

**Authors:** Ian L. Ross, Sofia Llahana, Michelle M. Anderson, Belene Demeke, Elouise M. Minnie, John A. H. Wass, Michelle Henry

**Affiliations:** ^1^ Department of Medicine University of Cape Town Cape Town South Africa; ^2^ School of Health and Medical Sciences, City St George's University of London London UK; ^3^ Department of Endocrinology Churchill Hospital Oxford UK; ^4^ Centre for Higher Education Development University of Cape Town Cape Town South Africa

**Keywords:** adrenal crisis, cross‐country comparison, glucocorticoid stress dosing, knowledge assessment, primary adrenal insufficiency, self‐management, vulnerability

## Abstract

**Objective:**

Adrenal crisis is a potentially fatal complication of primary adrenal insufficiency (PAI) and the leading cause of death in this population. Knowledge and its application are crucial to prevention. This study aimed to assess knowledge of glucocorticoid stress dosing and adrenal crisis prevention among individuals with PAI in South Africa (SA) and the United Kingdom (UK).

**Design:**

Cross‐sectional study comprising a researcher‐administered telephone survey in SA and an online questionnaire in the UK.

**Patients:**

A total of 286 individuals with PAI on glucocorticoid replacement therapy: 47 from SA and 239 from the UK.

**Measurements:**

The survey included questions on knowledge of glucocorticoid adjustment during intercurrent illness or stress, vulnerability (SA cohort only), and factors precipitating adrenal crises (UK cohort only).

**Results:**

Knowledge levels were suboptimal across both cohorts, with 72% answering correctly for infection, 54% vomiting, 52% fever, and 40% emotional stress. The UK cohort demonstrated significantly better knowledge (mean 49% correct, SD = 24.7) than the SA cohort (mean 34%, SD = 30.7; *p* < 0.001). Nearly a third of participants reported at least one adrenal crisis in the past year. Prior adrenal crisis experience correlated with higher knowledge scores (*p* = 0.049). Vulnerability scores correlated positively with adrenal crisis frequency in the SA cohort (*r* = 0.382; *p* = 0.008).

**Conclusions:**

This is the first study to compare knowledge of adrenal crisis prevention in individuals with PAI across two countries with distinct healthcare services, highlighting significant knowledge gaps in both cohorts, particularly in the SA cohort. Targeted education and support group engagement are needed to address knowledge gaps and reduce adrenal crisis incidence.

## 1 Introduction

Primary adrenal insufficiency (PAI), most commonly Addison's disease, is associated with impaired quality of life [1, 2], increased rates of sick leave, disability allowance and work loss [3, 4], reduced life expectancy [5], and predilection for developing infections [6]. Most patients with PAI require lifelong glucocorticoid replacement therapy, typically hydrocortisone tablets taken 2–3 times daily, and need to follow ‘sick day’ or ‘stress dosing’ rules during intercurrent illness, physical or emotional stress by increasing their glucocorticoid intake. In the event of an adrenal crisis, a life‐threatening complication often triggered by physiological stress such as infections, parenteral hydrocortisone is essential to prevent hospitalisation and death [7, 8, 9, 10, 11]. Approximately 6%–8% of patients with adrenal insufficiency experience an adrenal crisis each year [6, 12, 13], while 44% report at least one crisis since diagnosis [12, 14]. In South Africa, the reported adrenal crisis prevalence is 15% [15]. Mortality associated with an adrenal crisis is 0.5 per 100 patient‐years [12], despite deaths from adrenal crises potentially being preventable [7].

Effective self‐management and education of both patients with PAI and their families and caregivers are fundamental to improving health outcomes and preventing adrenal crises. This involves optimisation of daily glucocorticoid replacement, adherence to ‘sick day’ rules, and timely administration of parenteral hydrocortisone to prevent adrenal crisis. Patients are advised to double or increase their oral glucocorticoid dose during significant physiological stress such as fever with at least 40 mg hydrocortisone daily in two to four divided doses or 10 mg prednisolone daily in one to two divided doses until recovery. Patients are also advised to administer parenteral hydrocortisone and seek medical attention in situations where they are unable to take oral glucocorticoids such as continuous vomiting or diarrhoea [8, 11]. Preventative tools and strategies are also essential and include aspects such as having extra supply of oral glucocorticoids, carrying a steroid emergency card and hydrocortisone injection kit, wearing medical‐alert identification, and having the confidence and ability to self‐administer a hydrocortisone injection [8, 11, 16, 17].

Evidence suggests that 26%–38% of patients with adrenal insufficiency do not adjust the glucocorticoid dose during intercurrent illness [18, 19, 20]. Moreover, while over 70% of patients and caregivers are trained and have hydrocortisone injection kits [2, 21, 22], fewer than 25% manage to self‐inject or have this administered by a parent or caregiver [13, 22, 23, 24]. A recent US study reported that 41% of patients were unable to self‐inject despite attempting to do so during an adrenal crisis [25]. Immediate self‐injection of hydrocortisone is associated with a significantly lower rate of hospitalisation compared to waiting for healthcare professionals to administer hydrocortisone (38% vs. 73%; *p* = 0.008) [20], indicating a substantial benefit of self‐administration of hydrocortisone.

There are varied reasons why patients do not adhere to ‘sick day’ rules or fail to administer parenteral hydrocortisone in a timely manner. These may include insufficient knowledge of managing adrenal insufficiency, beliefs about the necessity of medication or the severity of illness, the complexity of using injectable hydrocortisone, and psychological or physical vulnerability, which is more prominent during an adrenal crisis [18, 20, 23, 26, 27]. In addition, some factors lie beyond patients' or caregivers' control, such as delays in emergency care, limited availability of medication, or a general lack of awareness of adrenal insufficiency among healthcare providers [21, 22, 23]. In some settings, barriers to effective self‐management may reflect both gaps in education and challenges in applying knowledge under duress. In South Africa for example, suboptimal assimilation of educational messages, compounded by systemic issues such as overcrowded community health centres and reduced consultation times [28], may constrain opportunities for effective patient education and hinder patients' ability to manage adrenal insufficiency effectively. These challenges contrast with European healthcare settings, where patients typically have more consistent access to patient education [11, 16, 29].

Vulnerability may also play a critical role. Vulnerable persons have a greater predisposition to physical and mental disease, and access healthcare more often than non‐vulnerable individuals [30, 31]. Vulnerability in this context primarily denotes predisposition to illness, suffering and mistreatment. Such vulnerability is often associated with psychosomatic symptoms, interpersonal difficulties, and mental distress. The severity and experience of vulnerability are subjective and shaped by individual perceptions. Vulnerability may induce physiological effects such as depression of the immune system, increasing susceptibility to infections, or psychological effects where individuals may feel helpless to exercise control over their health. It may also affect a person's sense of belonging and contribute to social isolation [31]. While it is reasonable to presume that patients with chronic, life‐threatening conditions such as PAI may experience heightened vulnerability, the extent to which this affects self‐management and adrenal crisis prevention has not previously been established.

We hypothesised that knowledge gaps and vulnerability contribute to patients' ability to prevent and manage adrenal crises. Specifically, we proposed that patients in the United Kingdom (UK) may demonstrate greater practical knowledge of PAI management than those in South Africa (SA), due to more consistent access to education and training. Our study aimed to assess the extent and predictors of knowledge among patients with PAI in preventing adrenal crisis, to examine differences for the first time between SA and UK cohorts, and to explore any possible associations between the degree of self‐reported knowledge and vulnerability, and adrenal crises.

## 2 Research Design and Methods

### 2.1 Ethics

Ethics approval was granted by the Faculty of Health Sciences Research and Ethics Committee at the University of Cape Town, SA, and the Health Research Authority in England, UK (IRAS ID 290622).

### 2.2 Study Design and Participants

We conducted a cross‐sectional study involving adult patients with PAI. This observational study followed the STROBE Statement [32] and utilised the STROBE Checklist for Cross‐sectional Studies to guide its design and reporting of the results.

In SA, a total of 15,672 emails were sent to endocrinologists, specialist physicians and general practitioners, asking them to invite their patients to participate. All potential participants were provided with a patient information sheet and informed consent. A total of 65 patients initially agreed to participate; of these, 10 could not be retraced, five subsequently declined, and three were excluded due to a diagnosis of secondary hypoadrenalism, leaving 47 patients who were enrolled. Following consent, a researcher‐administered telephone survey was conducted, with the researcher reading each question aloud and recording participants' responses.

In the UK, participants were recruited from the Addison's Disease Self‐Help Group (ADSHG), which has approximately 2000 members. Data were collected online using Qualtrics, an approved survey software, and disseminated via ADSHG membership email lists, website, newsletters, and social media. All participants were provided with a patient information sheet and completed an eligibility checklist based on self‐reporting to screen for inclusion. Completion of the anonymised survey implied consent. A total of 239 patients with PAI completed the survey, including 178 with Addison's disease.

Inclusion criteria:

- Adult patients aged 18 years or over;
- Diagnosed with PAI and on glucocorticoid replacement therapy at the time of study;
- Residing and receiving medical care either in South Africa or the United Kingdom.
  Exclusion criteria:
- Diagnosed with either secondary or tertiary adrenal insufficiency, or PAI caused by adrenal tumour.

### 2.3 Study Measures

The questionnaire included a series of questions relating to (a) demographics and clinical aspects of PAI, (b) knowledge of an adrenal crisis and its management, (c) a section on vulnerability (in the SA cohort only), and (d) a section on precipitating factors for adrenal crisis (in the UK cohort only). To assess knowledge, participants were asked how they had previously adjusted (or would adjust) their glucocorticoid dose during periods of increased stress or illness to prevent or manage an adrenal crisis. Eight statements relating to ‘stress dosing’ in situations such as fever, vomiting, surgical procedures, or emotional stress were presented, with each response coded as correct (1) or incorrect (0). The UK cohort selected from four predefined options in an online survey matrix: ‘no change in dose,’ ‘I take an extra hydrocortisone dose as required,’ ‘I double or triple the hydrocortisone dose,’ or ‘I inject hydrocortisone.’ For the SA cohort, responses were coded using the same matrix, based on participants’ answers to unprompted questions read by the researcher over the telephone. The option ‘I inject hydrocortisone’ was excluded, as not all participants had access to home emergency injection kits. In these cases, the response ‘I double or triple the hydrocortisone dose’ was considered correct for scenarios, such as vomiting and diarrhoea, where a UK participant would have selected ‘I inject hydrocortisone.’ The total knowledge score ranged from 0 to 8, with higher scores indicating greater knowledge.

Vulnerability was assessed in the SA cohort only using a validated questionnaire with binary ‘Yes/No’ responses for physical and psychological vulnerability, where the presence of more vulnerability features indicated greater severity. The presence of vulnerability was determined by enquiring as to the presence inter alia of anxiety, mood disturbance, social isolation, autonomic disturbances, physiological effects and ill‐health [33]. UK participants were asked to select the triggers of their past adrenal crises episodes from a predefined list, with the option to provide additional triggers through free‐text responses.

### 2.4 Statistical Analyses

Quantitative data were analysed using IBM SPSS, Version 28.0, with thresholds for statistical significance (α) set at 0.05. Descriptive data are presented as means and standard deviation, or as median and interquartile ranges for continuous variables, and frequencies and proportions for categorical variables. Independent sample *t*‐tests or Mann–Whitney–*U* tests compared continuous demographic and clinical variables between the SA and UK cohorts, while chi‐squared tests were used to compare categorical variables. Pearsons's correlations assessed the relationship between knowledge and number of crises, age, disease duration, hydrocortisone dose or number of comorbidities.

## 3 Results

### 3.1 Demographics and Clinical Information

A total of 286 patients with PAI completed the survey: 239 from the UK and 47 from SA, with a mean age of 47 years (SD = 14.8, range: 14–83 years). As illustrated in Table 1, the UK cohort comprised significantly more females (80%), compared with the SA cohort (60%); (*p* = 0.004). The median (interquartile range) duration of PAI was 10 years (IQR: 3–23) for the UK cohort, and 11 years (IQR: 5–23) for the SA cohort. The mean equivalent hydrocortisone dose was 21.5 mg, ranging from 10 to 50 mg in the SA cohort and 5–50 mg in the UK cohort. The most common hydrocortisone daily dose was 20 mg in both cohorts (SA: 39.5%, UK: 51.5%).

**Table 1:** Adrenal crises and comorbidities in adults with primary adrenal insufficiency from the South African and UK cohorts.

	Whole Cohort *N* = 286	SA Cohort *N* = 47	UK Cohort *N* = 239	*t/z/Χ* ^*2*^	*p*	*d/r/V*
Age, mean (SD)	47.3 (14.8)^d^	48.3 (16.9)	47.1 (14.4)^j^	−0.54	0.590	0.09
Sex, *N* (%)^a^				—n	0.004	0.19
Male	66 (23.1)	19 (40.4)	47 (19.7)			
Female	218 (76.2)	28 (59.6)	190 (80.2)			
Nonbinary	1 (0.3)	0	1 (0.4)			
Duration of illness, median years (IQR)	10 (3–23)	11 (5–23)	10 (3–23)	1.07	0.286	0.06
Had an adrenal crisis in the past year, *N* (%)	90 (33)^e^	15 (31.9)	75 (33.2)^k^	0.03	0.866	0.01
Number crises (past year), mean (SD)	0.6 (1.1)^e^	0.7 (1.5)	0.6 (0.9)^k^	−0.98	0.327	0.15
Equivalent hydrocortisone dose, mean (SD)	21.5 (6.92)^f^	21.9 (11.91)^i^	21.5 (5.60)	−0.45	0.651	0.08
Takes fludrocortisone, *N* (%)	241 (87.3)^g^	36 (76.6)	205 (89.5)^l^	5.88	0.015	0.15
Has emergency hydrocortisone injection kit, n (%)	226 (83.1)^h^	17 (36.2)	209 (92.9)^m^	89.01	< 0.001	0.57
Wears medic alert bracelet, *N* (%)	191 (70.2)^h^	23 (48.9)	168 (74.7)^m^	12.31	< 0.001	0.21
Comorbidities, median (IQR)	1 (0–2)	2 (1–3)	1 (0–2)	2.64	0.008	0.16
Reports comorbidities, *N* (%)^b^						
Mental health conditions	49 (18)	12 (28.6)	37 (16.1)	2.06	0.039	—
Metabolic and endocrine disorders	116 (42.6)	25 (59.5)	91 (39.6)	2.60	0.009	—
Respiratory conditions	22 (8.1)	5 (11.9)	17 (7.4)	1.07	0.285	—
Musculoskeletal disorders	23 (8.5)	5 (11.9)	18 (7.8)	0.95	0.342	—
Cardiovascular and circulatory conditions	23 (8.5)	4 (9.5)	19 (8.3)	0.34	0.728	—
Neurological disorders	18 (6.6)	3 (7.1)	15 (6.5)	0.21	0.834	—
Gastrointestinal conditions	26 (9.6)	6 (14.3)	20 (8.7)	1.22	0.222	—
Autoimmune and systemic disorders	20 (7.4)	4 (9.5)	16 (7)	0.66	0.509	—
Other health issues^c^	80 (29.4)	9 (21.4)	36 (15.7)	0.27	0.787	—

Abbreviations: IQR, interquartile range; SD standard deviation.

*Note:*

^a^ Data based on 238 patients in the UK sample.

^b^ Data based on 42 patients in SA cohort and 230 in UK cohort.

^c^ Includes kidney disease, visual problems, rheumatological, allergies, dermatological, and urological conditions.

^d^ Data based on 285 patients.

^e^ Data based on 273 patients.

^f^ Data based on 282 patients.

^g^ Data based on 276 patients.

^h^ Data based on 272 patients.

^i^ Data based on 43 patients.

^j^ Data based on 238 patients.

^k^ Data based on 226 patients.

^l^ Data based on 229 patients.

^m^ Data based on 225 patients.

^n^ Fisher's exact test.

In the SA cohort, 83% of patients were taking hydrocortisone and 17% prednisolone. In the UK cohort, 89% were taking short‐acting hydrocortisone, 6% prednisolone, 3% modified release hydrocortisone (Plenadren), and 2% were either on a hydrocortisone infusion pump or combination therapy such as short‐acting and modified release hydrocortisone. A higher proportion of the UK cohort (90%) compared with the SA cohort (77%) was taking fludrocortisone (*p* = 0.015). A higher proportion of the UK cohort (93%), compared with the SA cohort (36%) was in possession of an emergency hydrocortisone kit (*p* < 0.001) and wore a medic alert bracelet (75% vs 49%; *p* < 0.001); 67% (*N* = 159) of the UK patients were carrying the NHS emergency steroid card. While all UK participants were recruited via the Addison's Disease Self Help Group, only 21% (*N* = 10) of the SA cohort had ever been in contact with a patient support group.

A higher proportion of the SA cohort (*N* = 47) had comorbidities, with 96% of patients affected compared to 69% of the UK cohort (*N* = 239). Within the SA cohort, psychiatric conditions (*p* = 0.039) and metabolic and endocrine disorders (*p* = 0.009) were more common than in the UK cohort.

### 3.2 Adrenal Crises

Across both the UK and SA cohorts, 90 patients (33%) reported experiencing at least one adrenal crisis in the year preceding this study (Table 1). This equated to a mean of 0.6 (SD = 0.9) adrenal crises per patient in the UK cohort and 0.7 (SD = 1.5) in the SA cohort during the same period. Additionally, 101 patients (42%) in the UK cohort reported that their first adrenal crisis occurred at the time of diagnosis of adrenal insufficiency.

### 3.3 Knowledge to Prevent an Adrenal Crisis

Across both cohorts, most patients answered the question about glucocorticoid adjustment during an infection correctly (72%, *n* = 171). Fewer patients answered correctly for other scenarios: vomiting (54%, *n* = 122), fever (52%, *n* = 132), diarrhoea (51%, *n* = 115), stress (40%, *n* = 91), cold (36%, *n* = 97), dental procedures (36%, *n* = 97), and surgery (20%, *n* = 37).

**Figure 1:**
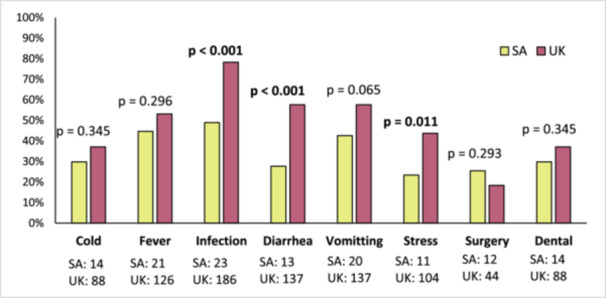
Comparison of knowledge scores between South African and UK cohorts.

Patients' responses to clinical scenarios requiring glucocorticoid adjustment were evaluated separately for the SA and UK cohorts. Participants in the UK cohort reported significantly better knowledge than their SA counterparts in the management of infection, diarrhoea, vomiting and situations of increased stress (Figure 1). However, in both cohorts, more than 50% of patients did not know how to respond appropriately to scenarios involving a cold, stress, surgery and dental procedures, and over 40% were unsure of the correct action in cases of diarrhoea and vomiting (Figure 1). Overall, the UK cohort demonstrated a higher mean knowledge score (*N* = 111, *M* = 48.9%, SD = 24.7%), representing an average of 3.9 out of 8 correct responses. In contrast, the SA cohort had a mean score of 34.0% (*N* = 47, SD = 30.7%), corresponding to an average of 2.7 out of eight correct responses, a difference that was statistically significant (*t* = 3.23, *p* < 0.001, *d* = 0.56). Notably, 32% of the SA cohort scored zero on the knowledge questionnaire compared to only 8% in the UK cohort.

**Figure 2:**
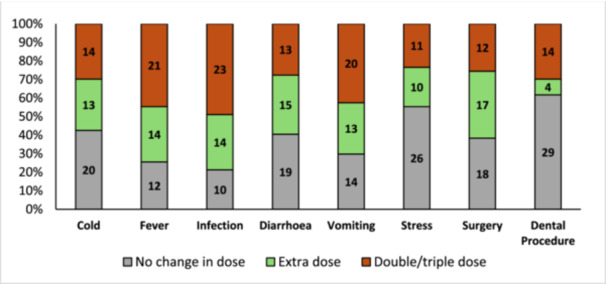
South African cohort responses on knowledge of glucocorticoid management during illness and stress.

In the SA cohort, nearly half of patients reported that they would correctly double or triple their glucocorticoid dose in cases of fever, infection, and vomiting; however, there was a general trend towards not adjusting their glucocorticoids in other scenarios (Figure 2).

**Figure 3:**
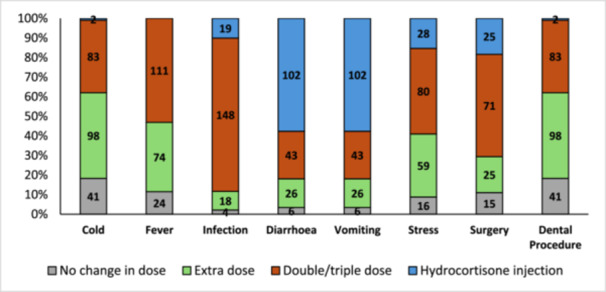
United Kingdom cohort responses on knowledge of glucocorticoid management during illness and stress.

In the UK cohort, most patients indicated they would double or triple their glucocorticoid dose during intercurrent infection and were most likely to administer a hydrocortisone injection in cases of diarrhoea and vomiting. However, an erroneous use of parenteral hydrocortisone was reported by patients in cases of intercurrent stress (15%) or surgical procedures (18%) (Figure 3).

### 3.4 Factors Influencing Knowledge of Adrenal Crisis Prevention

In both cohorts, patients with prior experience of an adrenal crisis reported significantly better knowledge than those who never had a crisis (49% vs 42%, *p* = 0.049), but no correlation was found between total knowledge score and number of crises. There was also a negative correlation between age and total knowledge score (*r* = −0.164; *p* = 0.040), indicating better knowledge in the younger patients. There was no correlation between, disease duration, hydrocortisone dose or number of comorbidities. In the SA cohort, knowledge was significantly better among patients who had accessed a patient support group, compared to those who never had contact with a support group (61% vs 27%; *p* < 0.001).

### 3.5 Triggers, Vulnerability and Association With Adrenal Crises and Knowledge

**Figure 4:**
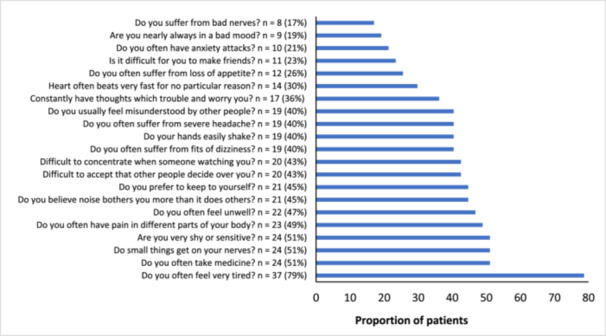
Proportion of patients in the South African cohort reporting vulnerability features (*n* = 47).

Participants in the UK cohort reported on triggers and circumstances surrounding their adrenal crises, with vomiting [45% (*N* = 102/227)] and diarrhoea [32% (*N* = 72/227)] being the most commonly reported precipitating factors; 22.5% of patients added additional triggers in free text such as exhaustion, dehydration, and ear infection, which were categorised as ‘others’ (Figure 4).

**Figure 5:**
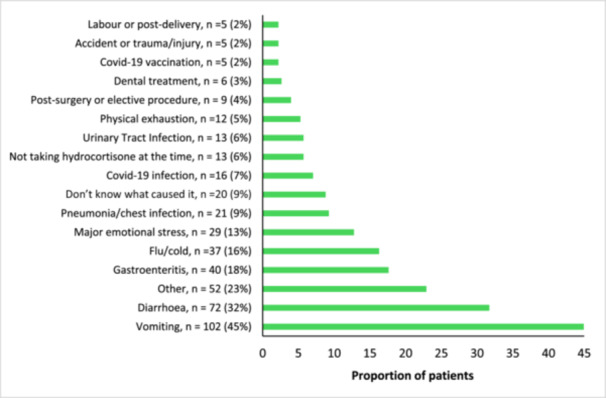
Triggers and precipitating factors for adrenal crisis in the UK cohort (*n* = 227).

Participants in the SA cohort reported on vulnerability factors, with the most commonly cited being fatigue (79%), the need to take medication (51%), irritability over small things (51%), and feelings of shyness or sensitivity (51%) (Figure 5). The total vulnerability score was positively correlated with the number of adrenal crises experienced in the previous year (*r* = 0.382; *p* = 0.008), but was not significantly correlated with knowledge score (*r* = 0.189, *p* = 0.203).

## 4 Discussion

This study included a diverse and clinically relevant sample of 286 individuals with primary adrenal insufficiency (PAI) across two distinct healthcare settings. While the UK and SA cohorts were similar in age and duration of diagnosis, notable differences emerged in sex distribution, treatment access, and knowledge of the condition and adrenal crisis management. The UK cohort had greater access to emergency hydrocortisone kits and medic alert identification and better overall knowledge of adrenal crisis prevention compared to their SA counterparts. Younger participants across both cohorts also reported great knowledge levels of their condition. Nevertheless, a significant proportion of participants across both cohorts reported suboptimal knowledge in addressing intercurrent illness and stress, diarrhoea, cold, fever, infection, vomiting, surgery or dental procedure, which could render them susceptible to an adrenal crisis. Notably, there was often erroneous use of parental hydrocortisone in the UK cohort in cases of intercurrent stress and surgery.

Nearly a third (33%) of participants in our study reported experiencing at least one adrenal crisis in the preceding year. When considering lifetime risk, Hahner et al. [12] and Li et al. [14] similarly found that 44% of patients with adrenal insufficiency had experienced at least one adrenal crisis since diagnosis, with a higher prevalence among patients with primary compared to secondary adrenal insufficiency (47% vs 41% [12] and 59% vs 31% [14], respectively. Although our study did not specifically assess lifetime adrenal crisis prevalence, the high proportion of patients reporting a crisis within a single year suggests a substantial burden over time. However, lifetime incidence estimates from patient self‐reported retrospective data may be subject to recall bias and often overestimate the true frequency of adrenal crises, particularly given the absence of a universally agreed definition. In contrast, prospective studies have reported lower incidence rates of approximately eight adrenal crises per 100 patient‐years [12, 13]. It is important to note here that these prospective studies were conducted in Germany, a healthcare setting distinct from the settings in our study, as standardised education programmes for patients with adrenal insufficiency and their caregivers have been successfully implemented [29]. Our combined cohorts demonstrated significant deficiencies in knowledge, which may have increased participants' vulnerability to an adrenal crisis. A perceived low need for additional hydrocortisone, along with concerns about glucocorticoid‐related adverse effects, may be obstacles to effective adrenal crisis management, contributing to reluctance to adopt stress dosing behaviours or to administer parenteral hydrocortisone in a timely manner [18, 23, 34, 35]. This highlights not only the importance of prospective studies to more accurately assess the true incidence and burden of adrenal crises, but also the urgent need for standardised patient education programmes across various healthcare settings.

Empowering patients and their caregivers in the use of stress doses of hydrocortisone has been associated with a reduction in adrenal crisis frequency [26]. In our survey, knowledge was found to be inadequate, particularly in the domains of managing a cold, diarrhoea, vomiting, stress, surgery and dental procedures, with over 50% of patients answering these questions incorrectly. Reinforcement of ‘sick day’ rules at every consultation or patient education session, through the provision of specific scenarios for glucocorticoid adjustment, is crucial to prevent escalation of illness or emotional stress that could precipitate an adrenal crisis, and may ultimately be lifesaving. Encouraging patients to maintain a diary documenting symptoms and occasions of ‘stress dosing’ could support ongoing individualised education and enhance shared decision‐making between patients and clinicians [19]. Education should be tailored to individual patient needs, emphasising appropriate and timely adjustment of oral glucocorticoids (stress dosing), adrenal crisis prevention, and addressing anxiety and misconceptions associated with glucocorticoid therapy and adrenal crisis management [9, 17, 36, 37].

Our study found notable differences between the two cohorts in access to preventative measures for adrenal crisis, with significantly fewer SA participants possessing an emergency hydrocortisone injection kit (36.2% vs 92.9%) or wearing a medic alert bracelet (48.9% vs 74.7%) compared to UK participants. While the recent NICE guidelines in the UK recommend that every patient with adrenal insufficiency is provided with an emergency hydrocortisone injection kit, this is not the case in SA where only a minority are offered emergency kits. To facilitate parenteral hydrocortisone administration, regular training for patients and caregivers should be provided at least annually either in person or online [8, 11, 16]. Our survey elicited a low threshold and sometimes inappropriate use of parenteral hydrocortisone in cases of intercurrent stress, which is not supported by guidelines and could be harmful in the long run. The complexity of the current hydrocortisone injection device further highlights the importance of regular patient education. Evidence suggest that, while most patients possessed an emergency injection kit, only 12%–24% managed to self‐inject or have this administered by a caregiver [13, 22, 23]; 42% identified the complex multi‐step process of the current hydrocortisone injection as the most significant barrier to self‐managing adrenal crisis [23].

Only 70% of patients in our study wore medical alert identification; this proportion was significantly lower in the SA cohort compared to the UK cohort (49% vs. 75%). This difference may reflect the impact of recent efforts by several European endocrine societies to raise awareness of adrenal crisis and to promote the use of standardised steroid emergency cards for children and adults, now available in more than 25 languages across Europe [11, 16, 38]. Provision of essential equipment, including steroid emergency cards, needles and syringes, parenteral hydrocortisone, and prescriptions for additional glucocorticoid tablets to support ‘stress dosing’ periods, is critical for effective self‐management. A recent US study found that 38% of participants reported difficulties with accessing needles and syringes, and in some cases, they were unable to self‐inject due to missing essential equipment [23].

In our combined cohort, knowledge was significantly better among patients who had previously experienced an adrenal crisis. We hypothesise that an adrenal crisis acts as an inflection point for patients, prompting more intensive education, improving symptom recognition, and encouraging earlier interventions, such as timely hydrocortisone injection or greater engagement with educational resources. This aligns with recent qualitative research suggesting that prior adrenal crises enhanced patients' ability to recognise symptoms and motivated them to be more proactive with their self‐management [35].

In our study, knowledge was also significantly better among patients who had contact with a patient advocacy group (PAG). While the entire UK cohort was recruited via a PAG, only 21% of patients in the SA cohort had contact with a PAG, and these individuals demonstrated higher knowledge levels compared to those without such contact. A survey of patients with rare endocrine conditions across several European countries found that 37% obtained information on managing their condition from PAGs, and 79% relied primarily on educational materials provided by these groups [39]. Our study did not assess the extent of education patients received from healthcare providers or the degree of engagement with support groups, which limits our ability to determine whether poor knowledge was due to limited interaction with PAGs, systemic healthcare limitations and access to patient education, or patient‐related factors such as memory and cognition issues, often reported by patients with PAI [40].

We were able to elicit that the majority of precipitants for an adrenal crisis in the UK cohort were predominantly gastrointestinal‐related and influenza, with vomiting being the pre‐eminent cause in 43% of patients, aligning with previous research [6, 13, 22, 23, 41]. Emotional stress was identified as a trigger in 12% of patients in our study, which is consistent with other studies [6, 22, 23].

Total vulnerability score was correlated with the number of crises. In the SA cohort we were able to elicit that patients felt most vulnerable with respect to feeling tired, having to take medication, and small things getting on their nerves. Vulnerability per se may induce heightened stress levels, exacerbate time pressures, induce lack of perspective, resulting in poor decision‐making. It results in an inability to plan and anticipate problems and may influence risk taking behaviour, potentially precipitating an adrenal crisis [42]. Considering that there is a high prevalence of vulnerable features among South African patients with PAI, it is plausible that UK patients with PAI may also demonstrate degrees of vulnerability, acknowledging that their adverse social circumstances may not exist to the same degree in the UK as in SA. We think that the presence of vulnerability may have a direct effect on the risk of adrenal crises per se due to enhanced susceptibility to infections and enhanced physiological demands. Moreover, vulnerability factors may play a role in reducing patients' ability to adapt to stressful circumstances by failing to adequately augment and adapt to their therapy in times of imminent crisis.

## 5 Conclusion

Our study highlights substantial deficiencies in patients' knowledge of glucocorticoid management in situations known to precipitate adrenal crises. This finding highlights the crucial role of clinicians in consistently reinforcing measures to prevent adrenal crises at every patient encounter. In South Africa, these results should prompt urgent action to prescribe and educate patients on the use of emergency hydrocortisone injection, while also supporting them to adjust their glucocorticoid doses appropriately during intercurrent illness and physical or emotional stress. These findings point to the need for structured, standardised education initiatives that are both practical and context‐specific, enabling patients adrenal insufficiency to manage their condition safely and effectively.

## Author Contributions

Ian L. Ross, Michelle Henry and Sofia Llahana conceptualised the study, developed the methodology, collected and analysed the data, and wrote the first manuscript draft. John A. H. Wass provided mentorship throughout the research. All authors contributed to manuscript revisions and approved the final version for submission.

## Conflicts of Interest

The authors declare no conflicts of interest.

## Data Availability

Datasets generated during the current study are not publicly available, but are available from the corresponding author on reasonable request.
